# The association between spatial access to physical activity facilities within home and workplace neighborhoods and time spent on physical activities: evidence from Guangzhou, China

**DOI:** 10.1186/s12942-020-00216-2

**Published:** 2020-06-20

**Authors:** Ye Liu, Xiaoge Wang, Suhong Zhou, Wenjie Wu

**Affiliations:** 1 School of Geography and Planning, Sun Yat-Sen University, Xingang Xi Road, Guangzhou, 510275 China; 2 Guangdong Key Laboratory for Urbanization and Geo-simulation, Sun Yat-Sen University, Xingang Xi Road, Guangzhou, 510275 China; 3 Guangdong Provincial Engineering Research Center for Public Security and Disaster, Guangzhou, 510275 China; 4 College of Economics, Jinan University, Guangzhou, 510632 China

**Keywords:** Physical activity facilities, Physical activity, Point of interest (POI), Home neighborhood, Work neighborhood, China

## Abstract

**Background:**

Urban residents from the developing world have increasingly adopted a sedentary lifestyle and spend less time on physical activities (PA). Previous studies on the association between PA facilities and individuals’ PA levels are based on the assumption that individuals have opportunities to use PA facilities within neighborhoods all day long, ignoring the fact that their willingness and opportunities to use nearby facilities depend on how much discretionary time (any time when people have a choice what to do) they have. Further, scant attention has been paid to the influence of PA facilities within both residential and workplace neighborhoods in the dense urban context. To address the above research gaps, this study investigated the links between the spatial access to PA facilities within home/workplace neighborhoods and time spent on PA among working adults, focusing on whether results were different when different measures of accessibility were used and whether participants’ discretionary time over a week affected their time spent on PA.

**Method:**

This study used data from a questionnaire survey (n = 1002) in Guangzhou between June and July 2017 and point of interest (POI) data from online mapping resources. Outcome variables included the amount of time spent on physical activity/moderate and vigorous intensity physical activity (PA/MVPA) over the past week. Home/workplace neighborhoods were measured as different distance buffers (500 m circular buffers, 1000 m circular buffers, and 1080 m network buffers) around each respondent’s home/workplace. Spatial access to PA facilities was measured using two indicators: the counts of PA facilities and proximity to PA facilities within home/workplace neighborhoods. The amount of discretionary time was calculated based on activity log data of working day/weekend day from the Guangzhou questionnaire survey, and regression models were used to examine relationships between the spatial access of PA facilities, the time spent on PA/MVPA, and the amount of discretionary time, adjusted for covariates. Associations were stratified by gender, age, education, and income.

**Results:**

Using different measures of accessibility (the counts of and proximity to PA facilities) generated different results. Specifically, participants spent more time on PA/MVPA when they lived in neighborhoods with more PA facilities and spent more time on MVPA when worked in closer proximity to PA facilities. A larger amount of discretionary time was associated with more time spent on PA/MVPA, but it did not strengthen the relationship between access to PA facilities and PA/MVPA time. In addition, relationships between access to PA facilities and PA levels varied by gender, age, education, and income.

**Conclusion:**

This study contributes to the knowledge of PA-promoting environments by considering both the home and workplace contexts and by taking into account the temporal attributes of contextual influences. Policymakers and urban planners are advised to take into account the workplace context and the temporal variability of neighborhood influences when allocating public PA facilities and public spaces.

## Background

Regular physical activity (hereafter, PA) provides many health benefits, including preventing overweight, improving mood, preventing coronary heart disease, promoting better sleep, lowering the risk of chronic disease such as stroke, high blood pressure, and type 2 diabetes [1, 2]. The World Health Organization recommended that adults should engage in at least 150 min moderate-intensity aerobic PA per week or at least 75 min vigorous-intensity aerobic PA per week to improve cardiorespiratory and muscular fitness and reduce the risk of non-communicable diseases [3]. However, an increasing proportion of Chinese people have adopted a sedentary lifestyle and have spent less time on occupation-, travel- and leisure-related PA over the past three decades due to urbanization and socioeconomic transition [4–7]. For example, an official report released in 2015 indicated that only 33.9% of Chinese people of all ages participated in PA regularly, and that only 14.7% of adults aged 20–69 did physical exercise regularly [8]. A growing body of literature has suggested that the neighborhood built environment influences residents’ willingness and ability to conduct PA, and that changing the neighborhood built environment may have an intervening effect on residents’ behaviors concerning PA [9–18]. Aspects of the built environment that shape residents’ PA levels include residential density [19], recreational facilities [20, 21], street connectivity [22], street intersection density [12], land-use mix [23, 24], access to transit [25], and urban greenery [26–32]. Among them, access to PA facilities (e.g., gyms, swimming pools, and soccer fields) is found to be closely related to residents’ engagement in PA, and to urban planners and designers, increasing the provision of PA facilities is thought to be an effective and straightforward way to increase residents’ PA levels [10, 33]. Thus, it is scientifically intriguing and practically important to unravel the link between spatial access to PA facilities and individuals’ engagement in PA, yet only limited efforts have been made to investigate this link in the Chinese high-density urban context.

Increasing the provision of PA facilities around individuals’ neighborhoods may lead to increased PA levels for the following reasons. First, spatial proximity from the home, workplace, or school to PA facilities means shorter travel times, lower travel costs, and less traffic-related stress. Indeed, proximal facilities can reduce people’s inconvenience of having to travel and increase their willingness to engage in PA [33, 34]. Second, people whose home, workplace, or school is near to PA facilities have more opportunities to view others doing PA in their daily life, and this will bring visual stimuli that arouse their interest in doing PA [35]. Thus, proximal PA facilities may have a stronger effect on people’s willingness to engage in PA, especially when doing exercise to stay healthy is a social norm [36]. Third, some PA facilities near the home, workplace, or school may serve as a convenient and accessible place where people can socialize with work colleagues, neighbors, or other students in the same school (for example, playing soccer with colleagues or neighbors on a nearby soccer field). People who wish to cement their relationships with those from the same neighborhood, work unit, or school may conduct group PA using nearby facilities.

Previous studies have evidenced that the counts of and proximity to PA facilities in the vicinity of people’s homes are positively associated with their engagement in PA [22, 28, 37, 38]. However, scant attention has been paid to the influence of PA facilities within workplace neighborhoods, where Chinese people at work spend much of their workday. Although a few researchers have investigated the effect of sport facilities in proximity to the workplace on PA behaviors in developed countries, such as the United States, the United Kingdom, and Japan [10, 33, 39], they are based on the assumption that people have the chance to use PA facilities within their neighborhoods all day long. In fact, individuals’ willingness and opportunities to use nearby facilities depend on the amount of discretionary time they have. Neglecting the temporal attributes of individuals’ surrounding environment may cause a bias in the estimation of the association between individuals’ PA levels and their access to nearby facilities.

The aim of this study is to investigate the links between spatial access to PA facilities in the vicinity of the home and workplace and time spent on PA among people living in Guangzhou, China. It particularly focuses on (1) the extent to which the counts of and proximity to PA facilities are associated with participants’ time spent on PA/MVPA; (2) whether the relationships vary by gender, age, education, and income; and (3) whether participants’ discretionary time over a week affects their time spent on PA/MVPA. It contributes to the knowledge of the PA-promoting effect of the built environment in the following respects. First, it takes into account both the home and workplace context when examining the association between spatial access to PA facilities and PA behaviors, thereby going beyond the conventional studies based on static residential contextual units. Second, it takes into account the temporal attributes of contextual influences (i.e., the amount of discretional time for PA activities) when examining the association between spatial access to PA facilities and PA levels. The findings of this study will help urban planners to allocate public PA facilities and to build a healthy city in an efficient and equitable manner.

## Method

### Study population

**Fig. 1:**
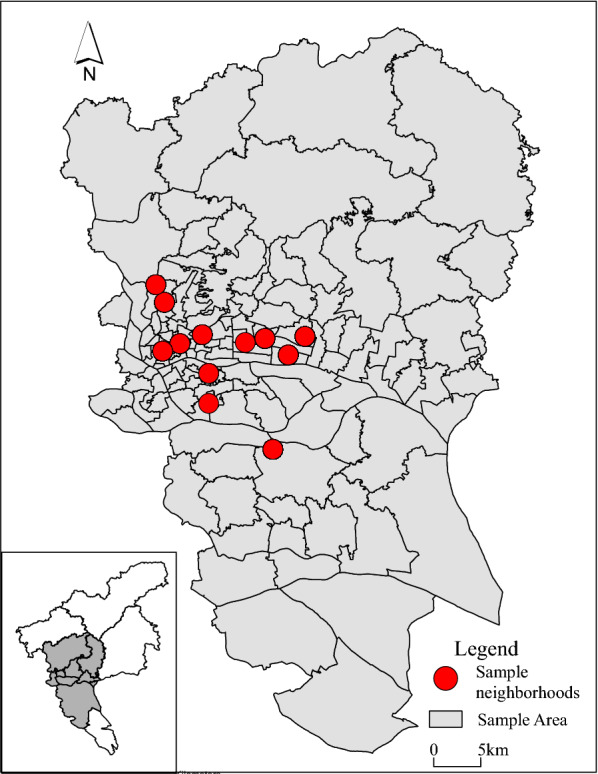
The location of sampled neighborhoods

We used data from a questionnaire survey carried out in Guangzhou between June and July 2017. We sampled 12 neighborhoods (with a mean area of 0.093 km^2^ and a mean population of 7665) from six inner-city districts of Guangzhou (Yuexiu, Tianhe, Baiyun, Liwan, Haizhu, and Panyu) using a multistage probability proportional to size (PPS) sampling technique (Fig. 1). The sample members (male = 49.95%, average age = 35.58, senior high school education = 60.82%, college or above = 32.80%) were representative of the general population of the six inner-city districts of Guangzhou (male = 51.36%, average age = 36.79, senior high school education = 61.23%, college or above = 30.76%, based on the official population statistics of the six districts of Guangzhou in 2015) [40], as the sampling was conducted rigorously. In each neighborhood, we used a systematic sampling approach to choose households randomly. We then invited one household member from each sampled household randomly to participate in a face-to-face interview. To qualify for the survey, respondents had to be aged between 18 and 60 years and not be students. The survey yielded a total of 1002 valid respondents. The response rate was 95.43%.

### Outcome

The outcome variables in this study included time spent on PA over the past week, time spent on MVPA over the past week. PA was classified into three intensity levels: light-intensity PA, moderate-intensity PA, and vigorous-intensity PA. Time spent on PA was gauged using the International Physical Activity Questionnaire – Short Form (IPAQ-SF) [41, 42].

#### Time spent on light-intensity PA

Two questions were asked to measure time spent on light-intensity PA: “How many days have you gone out for a walk (for recreational and relaxational purposes) for more than 10 min over the last week?” and “How many minutes on average did you spend on walking per day?” Time spent on light-intensity PA was calculated by multiplying the number of days spent on walking by the average time spent on walking per day.

#### Time spent on moderate-intensity PA

Two questions were asked to measure the time spent on moderate-intensity PA: “How many days have you participated in moderate-intensity PA (brisk walking, dancing, playing table tennis/badminton, bowling, etc.) for more than 10 min in the last week?” and “How many minutes on average did you spend on moderate-intensity PA per day?” Time spent on moderate-intensity PA was calculated by multiplying the number of days spent on moderate-intensity PA by the average time spent on moderate-intensity PA per day.

#### Time spent on vigorous-intensity PA

Two questions were asked to measure time spent on vigorous-intensity PA: “How many days have you participated in vigorous-intensity PA (aerobic fitness, running, fast cycling, swimming, etc.) for more than 10 min in the last week?” and “How many minutes on average did you spend on vigorous-intensity PA per day?” Time spent on vigorous-intensity PA was calculated by multiplying the number of days spent on vigorous-intensity PA by the average time spent on vigorous-intensity PA per day.

#### Time spent on PA/MVPA

Time spent on PA was the sum of time spent on light, moderate, and vigorous-intensity PA. Time spent on MVPA was the sum of time spent on moderate-intensity PA and time spent on vigorous-intensity PA.

### Predictors

We geocoded respondents’ home and workplace addresses using ArcGIS version 10.3. We generated the X and Y coordinates of home and workplace location points and then created 500 m circle buffers, 1000 m circle buffers, and 1080 m network buffers around each respondent’s home and workplace location point. The rationale of using 1080 m as the walking distance was that it took an adult around 15 min to walk 1080 m along the road network (at an average walking speed of 72 meters-per-minute), and an increasing number of Chinese urban planners used “15-minute walkable neighborhood” to assess residents’ access to urban facilities of all kinds [43, 44]. We used different types of buffers to alleviate the Modifiable Area Unit Problem (MAUP), which is a statistical bias in estimation arising from the use of different types of buffers.

#### Counts of PA facilities

We geocoded the addresses of PA facilities in Guangzhou using Point of Interest (POI) data from DDT Net for City Map (DDT is an acronym for “Dao Dao Tong”, which means “the road is clear”), a navigable electronic map provided by Guangdong Ruitu Wanfang Technology Co. Ltd. POI data were generated based on Baidu Map (a web mapping service application provided by Baidu) in 2016. The PA facilities in “DDT Map” included both classified facilities (e.g., basketball stadiums, tennis courts, swimming pools, soccer fields etc.) and unclassified facilities (e.g., multi-purpose stadia, gymnasiums and sports centers).

We used the number of PA facilities per 10,000 people within each respondent’s home buffer and workplace buffer (500 m circular buffer, 1000 m circular buffer, or 1080 m network buffer) to measure the count of PA facilities. Noted that 2015 one percent population sample survey data are released at the district level only, and it is impossible to access authoritative neighborhood population data around the time of Guangzhou survey. Therefore, we estimated population within each circular buffer by using high resolution (100 m) gridded population data provided by WorldPop (https://www.worldpop.org/). The WorldPop dataset used publicly available datasets (such as census data, land cover, building maps, and satellite nightlights) and Random Forest models to estimate population density at 100 m spatial resolution. The predicted number of people per 100 m grid cell in 2016 was estimated based on 2010 population census data and other datasets. The estimated population were then adjusted to match the official United Nations population estimates [45]. We calculated the total population within each home buffer or workplace buffer using the “Spatial Join” tool of ArcGIS version 10.3.

#### Proximity to PA facilities

We calculated the shortest distance between every respondent’s home and workplace address and the nearest PA facility. Road network distance was adopted in this regard. The road network data were also extracted from DDT Net for City Map.

#### The amount of discretionary time

Activity log data from the Guangzhou survey were used to measure the amount of discretionary time. The activity log recorded how respondents spent their time and what activities they had done over the last workday and the last weekend day, including traveling, subsistence activities (e.g., work and work-related study and training), personal affairs (e.g., sleeping, eating meals, doing personal care, seeing a doctor), family affairs (e.g., cooking, house-keeping, going grocery shopping, child care, caring for other family member), non-grocery shopping, relaxation and leisure (e.g., reading, watching TV, watching movies, physical exercise), social activities (e.g., connecting to others, visiting friends and family, going to party/banquet), and else. Given that subsistence activities, personal affairs, family affairs, and their associated travels are usually nondiscretionary (or even obligated), and some non-grocery shopping, social activities, and other activities were reported by respondents as non-discretionary activities, we calculated the amount of discretionary time on a workday (or a weekend day) by subtracting the amount of time spent on the above-mentioned activities (including all subsistence activities, all personal affairs, all family affairs, and their associated travels, and other activities that were considered as nondiscretionary by respondents themselves) on that day from 24 h [46]. The total amount of discretionary time over a week was computed as the amount of discretionary time over five working days plus the amount of discretionary time over 2 weekend days.

### Covariates

We adjusted for a series of covariates, following earlier studies of the relationships between PA behaviors and built environment. As individuals’ socio-demographic features have been proved to be associated with PA behaviors in previous studies [47, 48], the following socio-demographic covariates were taken into account: gender (male vs female), age (continuous variable), education (junior high school or below vs senior high school vs college or above), individual monthly income (continuous variable, in log), marital status (single vs married), hukou status (registered residency status in the household registration system, which is linked to one’s welfare entitlement; local non-agricultural hukou vs local agricultural hukou vs non-local non-agricultural hukou vs non-local agricultural hukou), car ownership (no car vs 1 or more cars), and average monthly neighborhood income per neighborhood resident (continuous variable, in log). Previous studies have found a positive relationship between community satisfaction and residents’ engagement in PA [49, 50]. Therefore, community satisfaction (satisfied vs neither satisfied nor dissatisfied vs dissatisfied) was included, and it was assessed based on respondents’ response to the question “Are you satisfied with the neighborhood where you are currently living?” The answer ranged from “strongly dissatisfied” to “strongly satisfied”.

We also considered two environmental covariates, namely the proportion of greenspace within each buffer (continuous variable), and intersection density within each buffer (continuous variable, in log). The area of greenspace was calculated using land cover data from DDT Net for City Map 2016. For the classification of land cover, greenspaces include forest, public park, and other types of greenspace. Intersection density (the number of junctions in each buffer) was used to measure street connectivity. This indicator was calculated using data from DDT Net for City Map 2016 as well. Previous studies have shown that higher street connectivity may represent a walking-friendly or a PA-friendly environment [11, 22].

### Statistical analyses

Linear regressions were run to estimate the association between time spent on PA/MVPA and spatial access to PA facilities. We divided the counts of PA facilities and the distance to the nearest PA facility into four quartiles and treated the first quartile as the referenced group. We applied a logarithmic transformation for the variable of the amount of discretionary time. For baseline models, we regressed outcome variables on predictors (with 500 m circular buffers) and covariates (Models 1–2). After that, we added interaction terms between the amount of discretionary time and access to PA facilities to test whether more discretionary time would strengthen the relationship between access to PA facilities and PA levels (Models 4–5).

To test the robustness of baseline models, we conducted several sensitivity analyses: (1) we replaced 500 m circular buffers with 1000 m circular buffers or 1080 m network buffers (Models 6–9) and reran the regressions; (2) we replaced time spent on PA/MVPA with time spent on indoor MVPA (as a proxy for time spent in PA facilities) as the dependent variable (Model 3); (3) we replaced the individual-varying contextual variables (such as the counts of and proximity to PA facilities, the proportion of greenspace, and intersection density) with the neighborhood-based contextual variables. Specifically, we generated new buffers around the centroid of respondents’ home neighborhoods rather than individuals’ home addresses and then generated new contextual variables based on the new buffers. In this case, respondents living in the same neighborhood will share the same variable of residential context. Stratified analyses were further conducted to explore the heterogenous effect of individuals’ demographic and socioeconomic characteristics on the association between PA behaviors and the attributes of PA facilities (Models 10–13). Variance inflation factors showed no evidence of multicollinearity among variables on the right-hand side. All analyses were conducted in STATA 14.0.

## Results

### Characteristics of study population

Table 1 summarizes the main characteristics of the study population. Respondents’ average time spent on PA was 131.32 min/week, with a SD of ± 111.18, and their average time spent on MVPA was 60.06 min/week, with an SD of ± 75.23. Only 11.57% respondents achieved the recommended amount of MVPA (150 min MVPA over a week). The number of PA facilities per 10,000 people in the home buffer was 6, 4, and 6 for 500 m circular buffer, 1000 m circular buffer, and 1080 m network buffer, respectively. The number of PA facilities per 10,000 people around the workplace were 4, 4, and 5, respectively. The mean distances from home/workplace address to the nearest PA facility were 155.62 m and 259.31 m, respectively. Respondents spent on average 710.38 min doing discretionary activities over a week.

**Table 1:** Descriptive Statistics of variables

Variables	Proportion/Mean (SD)
Outcome	
Time spent on PA (min)	131.32 (111.18)
Time spent on MVPA (min)	60.06 (75.23)
Achieved the recommended amount of MVPA (%)	11.57
Predictors	
Counts of PA facilities (500 m, home)	6 (4.596)
Counts of PA facilities (1000 m, home)	4 (3.042)
Counts of PA facilities (network, home)	6 (4.824)
Counts of PA facilities (500 m, workplace)	4 (9.522)
Counts of PA facilities (1000 m, workplace)	4 (5.314)
Counts of PA facilities (network, workplace)	5 (8.058)
Distance to PA facilities (home) (m)	155.62 (120.90)
Distance to PA facilities (workplace) (m)	259.31 (270.32)
Discretionary time over a week (min)	710.38 (542.77)
Gender (%)	
Male	49.95
Female	50.05
Age	35.58 (9.65)
Education (%)	
Junior high school or below	6.38
Senior high school	60.82
College or above	32.80
Individual monthly income (CNY)	6892.32 (3995.58)
Marital status (%)	
Single	19.94
Married	80.06
Hukou status (%)	
Local non-agricultural hukou	78.27
Local agricultural hukou	2.69
Non-local non-agricultural hukou	13.06
Non-local agricultural hukou	5.98
Car ownership (%)	
No car	63.81
1 or more cars	36.19
Greenspace proportion (500 m, home) (%)	0.75 (2.72)
Greenspace proportion (1000 m, home) (%)	0.45 (0.88)
Greenspace proportion (network, home) (%)	2.30 (2.36)
Greenspace proportion (500 m, workplace) (%)	1.75 (5.03)
Greenspace proportion (1000 m, workplace) (%)	3.37 (4.58)
Greenspace proportion (network, workplace) (%)	4.03 (4.34)
Average monthly neighborhood income per neighborhood resident (CNY)	15,637.20 (3266.61)
Community satisfaction	
Satisfied	80.96
Neither satisfied nor dissatisfied	15.75
Dissatisfied	3.29
Intersection density (500 m, home)	196 (76.97)
Intersection density (1000 m, home)	707 (267.68)
Intersection density (network, home)	670 (294.05)
Intersection density (500 m, workplace)	196 (71.15)
Intersection density (1000 m, workplace)	747 (264.02)
Intersection density (network, workplace)	721 (307.78)

Overall, respondents’ average age was 35.58 years old. Male respondents accounted for 49.95% of the total study population. About 6.38% of respondents had finished junior high school education or below only, 60.82% had finished senior high school education, and 32.8% had obtained a college education or above. The average monthly income of respondents was 6892.32 CNY. Respondents were over-represented in the categories of married (80.06%), local non-agricultural hukou holders (78.27%), those who were satisfied with their community (80.96%), and car non-owners (63.81%). The average monthly neighborhood income per neighborhood resident was 15,637.20 CNY. The average proportions of greenspace in the 500 m circular buffer, 1000 m circular buffer, and 1080 m network buffer around respondents’ home were 0.75%, 0.45%, and 2.30%, respectively, and those around respondents’ workplaces were 1.75%, 3.37%, and 4.03%, respectively. Intersection densities around the home were 196, 707, and 670 within the 500 m buffer, 1000 m buffer, and 1080 m buffer, respectively, and the corresponding intersection densities around respondents’ workplaces were 196, 747, and 721, respectively.

### The relationship between time spent on PA/MVPA, access to PA facilities, and discretionary time

Table 2 shows the result of associations between time spent on PA/MVPA and the attributes of PA facilities within 500 m buffers around respondents’ home/workplace. More time spent on PA/MVPA was significantly associated with more PA facilities per 10,000 people surrounding respondents’ home (Time spent on PA: Q2: Coef. = 74.946 SE = 13.660, Q3: Coef. = 24.063, SE = 14.281, Q4: Coef. = 50.863, SE = 11.227, respectively; Time spent on MVPA: Q2: Coef. = 25.068, SE = 10.093, Q3: Coef. = 19.425, SE = 10.447; Q4: Coef. = 21.237, SE = 7.920, respectively). Shorter distance to PA facilities surrounding respondents’ workplace increased the amount of time spent on MVPA (Time spent on MVPA: Q4: Coef. = -16.872, SE = 7.208). A larger amount of discretionary time was significantly linked to more time spent on PA/MVPA (Time spent on PA: Coef. = 6.596, SE = 1.343; Time spent on MVPA: Coef. = 3.527, SE = 0.919). There was no evidence to suggest that more discretionary time would strengthen the relationship between spatial access to PA facilities (counts or proximity) and time spent on PA/MVPA (Table 3).

**Table 2:** Regression on time spent on PA, 500 m circular buffer

	Model 1 Time spent on PA		Model 2 Time spent on MVPA		Mode l3 Time spent on indoor PA	
	Coef. (SE)		Coef. (SE)		Coef. (SE)	
Counts of PA facilities (Ref: Q1) (500 m, home)						
Q2	74.946***	(13.660)	25.068**	(10.093)	37.216***	(8.685)
Q3	24.063*	(14.281)	19.425*	(10.447)	2.806*	(9.316)
Q4	50.863***	(11.227)	21.237***	(7.920)	23.549***	(7.501)
Counts of PA facilities (Ref: Q1) (500 m, work)						
Q2	5.642	(9.801)	1.110	(7.566)	− 6.351	(5.708)
Q3	-4.286	(10.057)	-0.655	(7.216)	-1.286	(6.251)
Q4	2.035	(10.937)	0.626	(7.730)	− 1.636	(6.689)
Distance to PA facilities (Ref: Q1) (home)						
Q2	9.437	(12.896)	4.284	(8.771)	7.064	(7.965)
Q3	− 18.336**	(8.829)	− 1.245	(6.056)	− 7.652	(5.728)
Q4	63.922***	(12.235)	38.343***	(8.767)	14.244*	(7.820)
Distance to PA facilities (Ref: Q1) (work)						
Q2	− 5.051	(8.797)	− 1.902	(6.056)	− 1.296	(5.084)
Q3	2.401	(9.414)	0.510	(6.721)	1.926	(6.285)
Q4	− 16.936	(10.438)	− 16.872**	(7.208)	− 4.365**	(6.269)
Discretionary time over a week	6.596***	(1.343)	3.527***	(0.919)	1.631**	(0.770)
Sex (Ref: Female)						
Male	4.342	(7.034)	8.720*	(5.084)	4.579	(4.415)
Age	− 0.005	(0.028)	− 0.029***	(0.010)	− 0.011	(0.007)
Education (Ref: Senior high school)						
Junior high school or below	9.605	(10.219)	− 0.539	(7.511)	3.066	(8.694)
College or above	21.581***	(7.730)	14.984***	(5.396)	4.075	(4.989)
Income	25.011**	(11.311)	0.968	(7.307)	4.327	(5.990)
Marital Status (Ref: Married)						
Single/Divorced/Widowed	11.629	(8.881)	24.306***	(6.648)	2.593	(5.623)
Hukou Status (Ref: Local non-agricultural)						
Local agricultural hukou	1.616	(14.910)	3.452	(11.203)	12.298	(10.640)
Non-local non-agricultural hukou	− 9.096	(11.758)	− 1.257	(9.236)	1.386	(6.672)
Non-local agricultural hukou	− 9.023	(13.437)	− 9.801	(7.824)	7.868	(12.727)
Car ownership	0.132	(8.260)	5.170	(5.392)	− 0.321	(4.945)
Community Satisfaction (Ref: Satisfied)						
Neither satisfied nor dissatisfied	− 31.981***	(8.802)	− 8.050	(6.308)	− 12.322*	(6.344)
Dissatisfied	42.288**	(21.059)	16.295	(19.563)	− 9.201	(12.646)
Average monthly neighborhood income per neighborhood resident	− 15.847	(25.284)	− 1.022	(16.424)	− 37.815**	(14.764)
Greenspace proportion (500 m, home)	− 0.966	(0.849)	− 0.169	(0.915)	− 0.219	(0.659)
Greenspace proportion (500 m, work)	− 0.314	(0.526)	0.371	(0.414)	− 0.246	(0.386)
Intersection density (500 m, home)	− 0.038	(0.070)	0.022	(0.051)	− 0.036	(0.044)
Intersection density (500 m, work)	0.097*	(0.053)	0.008	(0.036)	0.004	(0.035)
Constant	− 32.832	(236.264)	2.281	(156.044)	338.519**	(144.960)
Observations	1002		1002		1002	
Adjusted *R*^2^	0.163		0.080		0.021	
AIC	12,124.745		11,438.358		11,235.205	

Robust Standard errors in parentheses; OR: odds ratio, 95% confidence intervals in parentheses; *p < 0.10, **p < 0.05, ***p < 0.01

**Table 3:** Interaction with access to PA facilities and discretionary time, 500 m circular buffer

	Model 4		Model 5	
	Time spent on PA		Time spent on MVPA	
	Coef. (SE)		Coef. (SE)	
Counts of PA facilities (home) (Ref: Q1) * Discretionary time				
Q2 * Discretionary time	− 0.364	(3.756)	3.832	(2.474)
Q3 * Discretionary time	− 3.686	(3.570)	− 4.345	(2.661)
Q4 * Discretionary time	1.639	(2.899)	− 0.525	(1.961)
Counts of PA facilities (work) (Ref: Q1) * Discretionary time				
Q2 * Discretionary time	− 0.495	(2.818)	− 0.204	(1.806)
Q3 * Discretionary time	1.919	(2.687)	− 0.468	(1.815)
Q4 * Discretionary time	2.592	(2.781)	1.314	(1.791)
Distance to PA facilities (home) (Ref: Q1) * Discretionary time				
Q2 * Discretionary time	− 4.133	(4.295)	− 0.998	(2.302)
Q3 * Discretionary time	− 3.688	(3.310)	− 3.329	(2.562)
Q4 * Discretionary time	8.519	(4.542)	6.294	(3.094)
Distance to PA facilities (work) (Ref: Q1) * Discretionary time				
Q2 * Discretionary time	1.081	(2.531)	− 0.346	(1.832)
Q3 * Discretionary time	− 3.046	(2.940)	0.066	(1.938)
Q4 * Discretionary time	0.470	(2.916)	− 1.072	(1.910)

Robust Standard errors in parentheses; *OR* odds ratio, 95% confidence intervals in parentheses; *p < 0.10, **p < 0.05, ***p < 0.01. All covariates in Table 2 have been adjusted for

### Sensitivity analyses

Table 4 reports the results of sensitivity tests based on PA facilities within different buffer zones (1000 m circular buffer and 1080 m network buffer). Consistent with the results from baseline models, more PA facilities per 10,000 people surrounding individuals’ home was significantly related to more time spent on PA/MVPA ((1000 m circular buffer: Time spent on PA Q2: Coef. = 57.609, SE = 16.946, Q3: Coef. = 87.670, SE = 16.007, Q4: Coef. = 54.390, SE = 17.905, respectively; Time spent on MVPA Q2: Coef. = 26.946, SE = 13.413, Q3: Coef. = 36.370, SE = 12.577, respectively); (1080 m network buffer: Time spent on PA Q2: Coef. = 28.593, SE = 30.231, Q3: Coef. = 11.011, SE = 31.069, Q4: 24.778, SE = 33.837, respectively; Time spent on MVPA Q2: Coef. = 13.100, SE = 17.138, Q3: Coef. = 2.436, SE = 16.847, Q4: Coef. = 15.595, SE = 19.971, respectively). The inverse relationship between time spent on PA/MVPA and distance from respondents’ workplace to the nearest PA facility was the strongest in the fourth quartile of distance to PA facilities (1000 m circular buffer: Time spent on PA: Coef. = -19.169, SE = 9.768, Time spent on MVPA: Coef. = -17.462, SE = 6.903; 1080 m network buffer: Time spent on PA: Coef. = -19.844, SE = 10.105, Time spent on MVPA: Coef. = -19.411, SE = 7.027). More discretionary time was still linked to more time spent on PA/MVPA (1000 m circular buffer: Coef. = 6.921, SE = 1.392; Coef. = 3.521, SE = 0.953; 1080 m network buffer: Coef. = 6.437, SE = 1.351; Coef. = 3.214, SE = 0.925).

**Table 4:** Regression on time spent on PA, 1000 m circular buffer and 1080 m network buffer

	Model 6 Time spent on PA_1000 m		Model 7 Time spent on MVPA_1000 m		Model 8 Time spent on PA_ network		Model 9 Time spent on MVPA_ network	
	Coef. (SE)		Coef. (SE)		Coef. (SE)		Coef. (SE)	
Counts of PA facilities (Ref: Q1) (1000m, home)								
Q2	57.609***	(16.946)	26.946**	(13.413)	28.593**	(30.231)	13.100*	(17.138)
Q3	87.670***	(16.007)	36.370***	(12.577)	11.011**	(31.069)	2.436**	(16.847)
Q4	54.390***	(17.905)	21.422	(13.829)	24.778**	(33.837)	15.595*	(19.971)
Counts of PA facilities (Ref: Q1) (1000m, work)								
Q2	− 1.651	(9.492)	− 6.248	(7.699)	− 13.286	(9.364)	− 8.882	(7.001)
Q3	− 14.015	(9.181)	− 17.108**	(7.197)	− 16.277	(10.092)	− 14.927**	(7.550)
Q4	− 0.259	(10.139)	− 7.082	(7.592)	2.323	(10.384)	− 7.157	(7.414)
Distance to PA facilities (Ref: Q1) (home)								
Q2	35.995***	(11.938)	15.567*	(8.049)	13.996	(9.403)	6.242	(6.243)
Q3	5.803	(8.772)	5.642	(5.968)	− 1.188	(13.366)	8.973	(8.793)
Q4	117.149***	(17.237)	54.975***	(13.396)	36.537	(31.350)	21.372	(17.519)
Distance to PA facilities (Ref: Q1) (work)								
Q2	− 8.549	(9.094)	− 2.759	(6.230)	− 7.990	(9.139)	− 2.949	(6.222)
Q3	1.783	(9.516)	− 1.042	(6.778)	4.651	(9.734)	0.121	(6.884)
Q4	− 19.169*	(9.768)	− 17.462**	(6.903)	− 19.844**	(10.105)	− 19.411***	(7.027)
Discretionary time over a week	6.921***	(1.392)	3.521***	(0.953)	6.437***	(1.351)	3.214***	(0.925)
Constant	− 1351.997***	(225.815)	− 479.798***	(150.511)	− 1044.623***	(271.524)	− 243.867	(175.542)
Observations	1002		1002		1002		1002	
Adjusted *R*^2^	0.160		0.086		0.148		0.080	
AIC	12,127.734		11,431.295		12,141.458		11,438.146	

Robust Standard errors in parentheses; *OR* odds ratio, 95% confidence intervals in parentheses; *p < 0.10, **p < 0.05, ***p < 0.01. All covariates in Table 2 have been adjusted for

Further sensitivity analyses were carried out to test the robustness of baseline models: 1) time spent on PA/MVPA was replaced by time spent on indoor MVPA as the dependent variable; 2) individual-varying contextual variables were replaced by neighborhood-based contextual variables. Results of these analyses showed no substantial difference from those of baseline models. Results of the first sensitivity analysis are reported in Table 2. Results of the second set of sensitivity analyses are available upon request.

### Stratified analyses by gender, age, education and income

Table 5 shows the results of stratified analyses with 500 m circular buffers around the home/workplace. For gender-stratified analyses, the relationship between time spent on PA and the counts of PA facilities around home in the second and the fourth quartiles was stronger for males than for females (Q2: Coef. = 55.747, SE = 19.057 for females; Coef. = 77.357, SE = 22.339 for males. Q4: Coef. = 42.499, SE = 16.083 for females; Coef. = 58.080, SE = 16.858 for males). The linkage between the amount of discretionary time and the counts of PA facilities around home was stronger for males than for females (Coef. = 5.374, SE = 1.814 for females; Coef. = 7.765, SE = 2.109 for males).

**Table 5:** Results of stratified analyses (Time spent on PA)

	Gender				Age			
	Model 10-1		Model 10-2		Model 11-1		Model 11-2	
	Female		Male		<35		≥35	
	Coef. (SE)		Coef. (SE)		Coef. (SE)		Coef. (SE)	
Counts of PA facilities (Ref: Q1) (500 m, home)								
Q2	55.747***	(19.057)	77.357***	(22.339)	57.709***	(20.212)	87.891***	(19.611)
Q3	26.251	(21.397)	12.012	(19.435)	30.755	(20.724)	24.119	(22.183)
Q4	42.499***	(16.083)	58.080***	(16.858)	44.100***	(15.242)	59.405***	(16.961)
Counts of PA facilities (Ref: Q1) (500 m, work)								
Q2	19.609	(13.628)	− 8.747	(13.605)	− 4.242	(13.964)	10.930	(13.824)
Q3	8.965	(13.441)	− 11.781	(15.281)	0.492	(15.092)	− 9.259	(14.042)
Q4	17.338	(15.155)	− 2.751	(15.799)	− 11.634	(15.309)	18.325	(16.374)
Distance to PA facilities (Ref: Q1) (home)								
Q2	13.353	(17.414)	15.923	(21.624)	16.737	(17.547)	2.314	(19.408)
Q3	− 15.619	(13.032)	− 13.968	(14.164)	3.266	(13.019)	− 37.379***	(12.168)
Q4	66.425***	(17.778)	66.692***	(18.592)	58.865***	(16.587)	81.080***	(18.073)
Distance to PA facilities (Ref: Q1) (work)								
Q2	4.523	(11.074)	− 20.192	(14.269)	5.941	(12.563)	− 15.825	(12.649)
Q3	12.827	(13.499)	− 8.531	(13.046)	− 6.141	(12.055)	11.498	(14.529)
Q4	− 15.152	(13.758)	− 19.096	(15.699)	− 7.858	(14.569)	− 23.472	(15.450)
Discretionary time over a week	5.374***	(1.814)	7.765***	(2.109)	6.595***	(1.825)	7.377***	(1.979)
Constant	− 235.745	(333.306)	20.042	(346.774)	117.054	(346.774)	− 163.816	(353.970)
Observations	502		500		519		483	
Adjusted *R*^2^	0.160		0.168		0.095		0.232	
AIC	6029.218		6114.577		6263.189		5877.565	

	Education				Income			
	Model 12-1		Model 12-2		Model 13-1		Model 13-2	
	Senior high education or below		College or above		< 6615 yuan		≥ 6615 yuan	
	Coef. (SE)		Coef. (SE)		Coef. (SE)		Coef. (SE)	
Counts of PA facilities (Ref: Q1) (500 m, home)								
Q2	32.287**	(16.092)	61.628*	(33.713)	47.091***	(17.179)	89.699***	(22.015)
Q3	15.980	(17.195)	42.561	(39.594)	29.226*	(17.441)	− 13.031	(26.017)
Q4	42.634***	(13.222)	79.961***	(28.485)	37.317***	(13.806)	90.695***	(23.266)
Counts of PA facilities (Ref: Q1) (500 m, work)								
Q2	15.407	(10.317)	− 3.399	(21.499)	4.401	(11.071)	5.867	(18.840)
Q3	2.752	(10.863)	− 3.016	(22.068)	0.900	(11.898)	− 7.161	(18.059)
Q4	3.629	(11.038)	13.254	(22.766)	− 4.564	(13.073)	15.432	(19.725)
Distance to PA facilities (Ref: Q1) (home)								
Q2	31.796**	(13.878)	32.483	(43.653)	16.913	(14.933)	29.146	(28.828)
Q3	− 11.391	(9.444)	6.941	(29.227)	− 11.338	(9.705)	− 13.590	(18.144)
Q4	71.068***	(13.845)	85.444**	(39.789)	64.369***	(14.722)	111.387***	(28.161)
Distance to PA facilities (Ref: Q1) (work)								
Q2	− 7.535	(8.826)	− 2.017	(20.375)	3.662	(10.856)	− 19.463	(15.743)
Q3	− 7.880	(9.331)	21.630	(21.420)	− 7.157	(10.312)	24.921	(18.606)
Q4	− 7.416	(10.831)	− 30.329	(22.315)	− 22.093*	(12.487)	− 0.822	(20.451)
Discretionary time over a week	5.733***	(1.350)	8.409**	(3.244)	6.767***	(1.698)	6.797***	(2.177)
Constant	− 266.578	(271.191)	213.659	(479.641)	− 132.641	(305.727)	259.063	(394.270)
Observations	673		329		628		375	
Adjusted *R*^2^	0.107		0.213		0.115		0.206	
AIC	7894.841		4143.823		7463.670		4650.258	

Robust Standard errors in parentheses; *OR* odds ratio, 95% confidence intervals in parentheses; *p < 0.10, **p < 0.05, ***p < 0.01. All covariates in Table 2 have been adjusted for

We used 35 years old as the threshold for age-stratified analyses, because the average age of participants in this study was 35.58. The relationship between time spent on PA and the counts of PA facilities around home was stronger for the older group than for the younger group (Q2: Coef. = 57.709, SE = 20.212 for age < 35; Coef. = 87.891, SE = 19.611 for age ≥ 35. Q4: Coef. = 44.100, SE = 15.242 for age < 35; Coef. = 59.405, SE = 16.961 for age ≥ 35). The association between the amount of discretionary time and the counts of PA facilities around home was stronger the older group than for the younger group (Coef. = 6.595, SE = 1.825 and Coef. = 7.377, SE = 1.979, respectively).

For education-stratified analyses, the relationship between time spent on PA and the counts of PA around home was stronger for the higher-educated than for the lower-educated (Q2: senior high education or below: Coef. = 32.287, SE = 16.092; college or above: Coef. = 61.628, SE = 33.713. Q4: senior high education or below: Coef. = 42.634, SE = 13.222; college or above: Coef. = 79.961, SE = 28.485). The association regarding discretionary time was stronger the higher-educated than for the lower-educated was well (Coef. = 5.733, SE = 1.350; Coef. = 8.409, SE = 3.244, respectively).

We used 6615 yuan as the threshold for income-stratified analyses, because the average monthly income in Guangzhou was 6615 in 2017. The relationship between time spent on PA and the counts of PA around home was stronger for the higher-income group than for the lower-income group (Q2: Coef. = 47.091, SE = 17.179, Q4: Coef. = 37.317, SE = 13.806 for the lower-income group. Q2: Coef. = 89.699, SE = 22.015; Q4: Coef. = 90.695, SE = 23.266 for the higher-income group). The linkage between distance to PA facilities and time spent on PA was significant for the lower-income group only (Coef. = -22.093, SE = 12.487). The association regarding time spent on PA was nearly the same for both income groups (Coef. = 6.767, SE = 1.698; Coef. = 6.797, SE = 2.177, respectively).

## Discussion

This study contributes to our understanding of the links between individuals’ spatial access to PA facilities and their PA engagement by considering both home neighborhoods and workplace neighborhoods. As some office workers often go to the place of exercise directly from their workplace rather than their home (for example, 25% working participants in our survey reported that their usual place of physical exercise was more proximal to their place of work than to their home), taking into account PA facilities around individuals’ worksite enable researchers to better capture the PA-promoting environments to which they are exposed in their daily activities. Moreover, this study takes into account the temporal characteristics of contextual influences when examining the association between access to PA facilities and PA engagement. It is assumed that those with more discretionary time are able to spend more time on PA than those working around the clock. In addition, the association between PA facilities and PA behaviors is supposed to vary by individual characteristics, such as gender, age, education level, and income.

In the current study, we found that using different measures of accessibility to PA facilities (i.e., counts and proximity) would generate different results. A significant association between the counts of PA facilities around the home and the amount of time spent on PA/MVPA can be found in the cities of some developed countries [51–53]. For example, a Norwegian study found that having more sports fields in the home neighborhood was related to children’s PA levels [52], while a New York study found that the counts of PA facilities around the home was associated with PA levels, especially for people who had gym membership [51]. In addition, a US study found that the density of commercial PA facilities was related to PA behaviors among U.S. teenagers [53].

Regarding the other measure of spatial accessibility (proximity), our study found no evidence that living closer to a PA facility increased individuals’ PA levels. A possible explanation is that most respondents’ home address is very close to at least one PA facility (the average distance is 155.62 m, and 99.8% respondents fall into the range of 0 to 500 m), and people usually do not mind walking a longer distance for sports within such a short distance. Another explanation is that some people may travel to somewhere in the city to do team sports (e.g., soccer and basketball) and racket sports (e.g., tennis and badminton) with their colleagues and friends living in other neighborhoods. The third explanation is that our models do not adjust for some covariates related to attitude toward regular PA. Respondents who are socioeconomically advantaged (and who are more likely to do PA regularly) tend to live in large real estate neighborhoods, and those who are socioeconomically disadvantaged tend to live in inner-city traditional neighborhoods and informal housing neighborhoods (e.g. villages in the city). The second types of neighborhoods generally have better access to PA facilities than the first type of neighborhoods. This finding is consistent with evidence from the United Kingdom and France [54, 55]. Specifically, a study on UK adults reported no association between engagement in PA and residential proximity to sports centers [54]. However, a study conducted in Paris generated a more complicated result: the likelihood of practicing three types of sports (team sports, racket sports, and fitness) was not linked to residential proximity to the corresponding sports facilities, but the likelihood of swimming and related activities was related to spatial accessibility to swimming pools [55].

This study found no significant positive relationships between the time spent on PA/MVPA and the counts of PA facilities around the workplace, which was consistent with the results from three studies. The first study, conducted in Aichi, Japan, found no relationship between the counts of sport facilities around the workplace and leisure-time habitual walking/exercise [56]. The second study, carried out in the Seattle area in the United States, found that a worksite’s fitness destinations were not related to MVPA during leisure time, probably because most respondents had limited time to do PA on a weekday [20]. The third study, carried out in West Central Scotland, indicated that PA frequency was not associated with the counts of PA facilities around the workplace [33]. Adlakha et al. study, conducted with participants in four US metropolitan areas, also found that recreation facilities around the workplace were not related to the possibility of meeting the recommended levels of total PA. However, when domain-specific PA (work, travel, and leisure) were considered, recreation facilities in the workplace neighborhood were significantly related to time spent on travel PA and leisure PA [10].

In the current study, a closer workplace proximity to PA facilities appeared to be linked to higher PA or MVPA levels, while residential proximity to PA facilities had no effect. An explanation is that people tend to use the closest facility for PA after work due to time constraints, and they tend to do PA alone or with their work colleagues (who also prefer the closest facility for team sports or racket sports) on a workday. Another reason is that respondents’ workplace address is farther away from PA facilities than their home address (the average distance is 259.31 m, and more than 20% respondents fall into the range of 500 m–4000 m). In this case, people tend to be reluctant to use a PA facility outside their workplace neighborhood (e.g. > 500 m) and to do PA on a workday.

Regarding the association between discretionary time and PA behavior, we observed that those with more discretionary time spent more time on PA. The last three decades have witnessed a substantial decline in PA levels and a dramatic increase in time spent on sedentary behaviors among Chinese people [7, 53, 57, 58]. One reason behind this trend is that Chinese working-aged people are working longer hours than ever before, and the proportion of more sedentary occupations has become increasingly prevalent over time [7, 53]. Another reason is that domestic PA levels have declined over time among Chinese people, especially Chinese women [53]. Under such circumstances, those who spend less time on work and family affairs would have more time for relaxation and leisure, including leisure time PA. We also found that more discretionary time would not strengthen the association between spatial access to PA facilities (in this case, the counts of facilities and distance to PA facilities in both residential neighborhoods and workplace neighborhoods) and PA behaviors.

Consistent with previous studies [48, 59], the results of stratified analyses showed that the links between spatial access to PA facilities and time spent on PA varied by demographic and socioeconomic characteristics. In the current study, men’s time spent on PA was more related to the counts of PA facilities around home than women’s time spent on PA. One possible explanation is that men are more inclined than women to do physical exercise that requires PA facilities (e.g. team sports, racket sports and fitness). The older cohort’s PA time (aged 35 or above) was more related to the counts of PA facilities around home than the younger cohort’s PA time. Old working-aged adults spent more time at home with their family and children than younger working-aged adults in the contemporary China. Thus, the former group more influenced by PA-promoting environment around home than the latter group. Consistent with previous studies, higher-educated and higher-income respondents’ PA time was more related to the counts of PA facilities around home than lower-educated and lower-income respondents’ PA time [55, 60, 61]. One explanation is that higher-educated and higher-income people are more aware of health than their lower-educated and lower-income counterparts, and the former group has a stronger willingness to use PA facilities than the latter group. Another explanation is that the former group has a stronger ability to pay than the latter group. Interestingly, spatial proximity to PA facilities around workplaces mattered to the lower-income group only. This may be because lower-income people are more sensitive about distance constraints than higher-income groups, especially in terms of locations of PA facilities around their worksite.

This study has some limitations. Firstly, we were not able to assess whether there was a causal relationship between access to PA facilities and PA behaviors, as our data were cross-sectional. Secondly, the indicators of time spent on PA and the intensity of PA in our research were generated based on self-reported data, which may lead to self-report bias. Further research is needed to verify the results by using objective and more accurate data on PA (for example, data from accelerometers and global positioning system detectors). Thirdly, we did not take into account the types of PA facilities (e.g., public versus private, paid versus free, for different physical exercises) in the current study, which had been found to influence people’s willingness to do PA [33, 35, 55]. For example, a study conducted in West Central Scotland found that individuals’ PA frequency was higher where they lived in closer distance to private PA facilities than the public PA facilities [33]; a San Diego study found that residents’ exercise habits was significantly related to the density of paid PA facilities but not to the density of free and public PA facilities [35]; a French study found that spatial accessibility to swimming pools was related to time spent on swimming and related exercise, but that spatial accessibility to other types of facilities (team sport, racket sports, and fitness) was not associated with the practice of other sports [55].

Fourthly, we were not able to distinguish between types of PA (e.g., work-, travel- and leisure-related PA) and between types of leisure-related PA (e.g., jogging, swimming, racket sports), as the Guangzhou survey used a short version (generic items) instead of a long version (activity domains asked independently) of IPAQ. Besides, we were not able to differentiate between time spent on PA on the workday and on the weekend, as the short version of IPAQ recorded the total amount of time spent on PA over a week. Fifthly, individual preferences and hobbies are included in our analysis, as related variables are missing in our dataset. Noted that people who are fond of sports are more influenced by the counts of and proximity to PA facilities within their neighborhood than sedentary people. Sixthly, time spent on PA does not necessarily imply the actual usage of PA facilities, and some respondents may do physical exercise outside PA facilities. Seventhly, model results may be biased due to the clustering of respondents’ residential addresses. Noted that only twelve residential neighborhoods are sampled in Guangzhou survey, and addresses in the same residential neighborhood are close to each other. Finally, residential selection bias might lead to overestimation in the linkage between built environment and PA intensity. For example, sporty individuals tend to choose to live in neighborhoods with better access to PA facilities, and they perform physical exercise more often than others.

## Conclusions

This study examines the associations between access to PA facilities within both residential and workplace neighborhoods and individual’s time spent on PA/MVPA in the Chinese high-density urban context. Our research shows that more PA facilities within residential neighborhoods are associated with more time spent on PA/MVPA, and closer proximity to the workplace to PA facilities is linked to more time spent on PA/MVPA. A larger amount of discretionary time leads to more time spent on PA/MVPA but does not strengthen the relationship between access to PA facilities and PA/MVPA time. In addition, relationships between access to PA facilities and PA levels vary by gender, age, education, and income.

Policymakers and urban planners are advised to take into account the workplace context and the temporal variability of neighborhood influences when allocating public PA facilities and public spaces, instead of focusing solely on the static residential context. Specifically, public sports facilities planning in some countries (including China) normally use the number of facilities per ten thousand resident population (*wan ren zhi biao*) to assess the provision of sports facilities within an area. Planners can use statistics based on working population to assess the provision of facilities and to locate sports facility-deficient areas in the future. The availability of mobile big data (cell phone data and Baidu Huiyan data, for example) nowadays enables planners to obtain real-time population statistics across the entire city. Furthermore, given that individuals’ willingness to conduct PA is constrained by the amount of their discretionary time in a day, planners are advised to make public PA facilities close to other destinations (e.g. grocery stores, schools, libraries, and restaurants) in the daily life. For example, large PA facilities and other destinations are encouraged to concentrate in a particular area through the practice of zoning. Property developers which are involved in urban redevelopment are required to provide a certain amount of publicly accessible space for PA within the land plot. People living in a neighborhood with better destination accessibility will have more discretionary time and thus are more willing to do PA.

## Data Availability

The datasets used and/or analysis during the current study are available from the corresponding author on reasonable request.

## Abbreviations

PA: Physical activity
MVPA: Moderate-intensity and vigorous-intensity physical activity
POI: Point of interest
Coef: Coefficient
SE: Standard error
OR: Odds ratio
95% CI: 95% confidence interval
